# Posterior Interosseous Artery Flap: Anatomy, Applications, and Outcomes

**DOI:** 10.7759/cureus.109906

**Published:** 2026-05-29

**Authors:** Maradona M Mashigo, Lebohang Mookamedi, Obakeng Jasson, Grant Biddulph

**Affiliations:** 1 Orthopaedic Surgery, University of the Witwatersrand, Johannesburg, ZAF; 2 Plastic and Reconstructive Surgery, University of the Witwatersrand, Johannesburg, ZAF; 3 Orthopaedic Surgery, Job Shimankana Tabane Hospital, Rustenburg, ZAF; 4 Orthopaedic Hand Surgery, University of the Witwatersrand, Johannesburg, ZAF

**Keywords:** dorsal hand defects, forearm flap, hand reconstruction, perforator flap, pia flap, posterior interosseous artery flap, soft tissue defects, upper limb reconstruction

## Abstract

The posterior interosseous artery (PIA) flap remains an important regional reconstructive option for soft tissue defects of the hand and wrist because it provides thin, pliable tissue while preserving the major vascular axes of the forearm. This narrative review discusses the anatomical basis, surgical technique, indications, outcomes, complications, and comparative role of the PIA flap in contemporary upper limb reconstruction. Current literature demonstrates generally favorable functional and aesthetic outcomes with reliable soft tissue coverage, particularly for defects involving the dorsal hand, wrist, thumb, and first web space. Although venous congestion and anatomical variability remain recognized limitations, careful surgical planning and technical modifications may reduce complication rates. Compared with alternative regional and free flap options, the PIA flap continues to serve as a valuable vessel-preserving reconstructive technique, particularly for moderate-sized defects requiring durable coverage and satisfactory functional recovery. However, the available evidence is derived predominantly from retrospective case series and observational studies, highlighting the need for prospective comparative studies to further define its role in contemporary upper limb reconstruction.

## Introduction and background

Soft tissue defects of the hand and wrist remain a significant reconstructive challenge due to the thin soft tissue envelope and the need to preserve both function and aesthetic integrity. Even relatively minor trauma can expose underlying tendons, nerves, and bone, often necessitating flap-based reconstruction rather than simple grafting techniques [1,2]. Contemporary reconstructive approaches increasingly favor local and perforator-based flaps, which provide well-vascularized, thin, and pliable tissue with reduced donor-site morbidity and improved functional outcomes [3-5].

The posterior interosseous artery (PIA) flap has emerged as a reliable regional option for reconstruction of defects involving the dorsum of the hand, wrist, and first web space. Its principal advantage lies in preserving the major axial vessels of the forearm while maintaining a consistent vascular pedicle, a favorable arc of rotation, and good tissue match [3,4,6]. However, its successful application depends on a detailed understanding of vascular anatomy, particularly perforator distribution and distal anastomoses, as complications such as venous congestion and partial flap necrosis remain recognized limitations [4-6], with advances in anatomical and imaging studies improving preoperative planning and surgical precision [5,7]. The objective of this narrative review is to provide a comprehensive overview of the anatomical basis, surgical technique, indications, outcomes, complications, and contemporary applications of the PIA flap in upper limb reconstruction.

## Review

Literature search strategy

A narrative literature review was conducted using PubMed and Google Scholar. Articles published between January 2008 and March 2026 were searched using combinations of the keywords "posterior interosseous artery flap", "posterior interosseous flap", "reverse posterior interosseous artery flap", "hand reconstruction", "wrist reconstruction", "forearm flap", and "upper limb soft tissue reconstruction". Relevant anatomical studies, clinical case series, comparative studies, and review articles published in English were included. Reference lists of selected articles were also manually reviewed to identify additional relevant publications. The included literature was used to provide a comprehensive overview of the anatomy, surgical technique, indications, outcomes, complications, and contemporary applications of the PIA flap.

Anatomy

The posterior interosseous artery (PIOA) arises from the common interosseous artery, typically a branch of the ulnar artery, and passes through the interosseous membrane to enter the dorsal forearm, emerging beneath the supinator muscle approximately 15 to 18 cm proximal to the ulnar styloid [4,8]. It then courses distally within the intermuscular septum between the extensor carpi ulnaris and extensor digiti minimi muscles, closely related to the posterior interosseous nerve. This anatomical relationship is of surgical importance due to the risk of nerve injury during flap elevation and the presence of a vascular plexus supplying the nerve [9,10].

Along its course, the PIOA gives rise to multiple septo-cutaneous perforators, typically four to eight, predominantly located in the middle third of the forearm, forming a reliable supra-fascial vascular network for fascio-cutaneous flap elevation [4,6]. Distally, it forms a critical anastomosis with the anterior interosseous artery approximately 3-5 cm proximal to the distal radioulnar joint, which provides the anatomical basis for reverse-flow flap perfusion [4,6]. The dorsal forearm is further supported by a rich anastomotic network involving the anterior interosseous, posterior interosseous, radial, and ulnar arteries, contributing to robust cutaneous perfusion and flap reliability [5,7].

Recent cadaveric studies have demonstrated that the PIA most commonly originates from the common interosseous artery and provides a reliable reverse-flow pedicle with consistent perforator distribution in the middle third of the forearm [11]. Additional anatomical studies have highlighted the close relationship between the PIA, interosseous recurrent artery, and posterior interosseous nerve, emphasizing the importance of careful septal dissection during flap harvest [12]. Detailed surgical anatomical descriptions have further demonstrated the relationship of the vascular pedicle with the extensor digiti minimi, extensor carpi ulnaris, and branches of the posterior interosseous nerve during flap elevation [13]. Rare anatomical variations, including absent distal anastomoses, hypoplastic vessels, and congenital absence of the PIA, have also been described in the literature, reinforcing the importance of careful preoperative vascular assessment [13,14].

The increasing use of preoperative vascular imaging has further improved the reliability and safety of PIA flap harvest [7,11]. Handheld Doppler ultrasonography remains the most widely used and accessible method for identifying septo-cutaneous perforators and confirming distal arterial communication before surgery [7,13]. More advanced imaging modalities, including computed tomography angiography (CTA) and three-dimensional vascular reconstruction, have demonstrated value in accurately mapping perforator distribution, pedicle calibre, and anatomical variations, thereby improving flap planning and reducing intraoperative uncertainty [4]. Recent imaging studies using three-dimensional CTA fusion imaging have shown high accuracy in perforator localization and demonstrated the ability to provide continuous visualization of both perforators and the vascular pedicle, particularly in traumatized extremities where conventional imaging may be limited [7]. Detailed anatomical mapping studies have similarly confirmed the reliability of perforator distribution in the middle third of the forearm while emphasizing the importance of defined surgical landmarks during flap design [4,6,11].

In addition to arterial anatomy, venous drainage plays a critical role in flap viability and remains an important consideration during flap elevation [6,15]. The venous outflow of the PIA flap is primarily mediated through venae comitantes accompanying the vascular pedicle together with the subdermal venous plexus [6,12]. Venous congestion remains one of the most frequently reported complications of the flap and may occur secondary to pedicle compression, excessive tunnelling, tension, or injury to the superficial venous network during dissection [6,15]. Adequate venous drainage is essential for flap survival and may be influenced by preservation of the superficial venous network during flap elevation. Venous return occurs through interconnected superficial and deep venous channels, including the venae comitantes accompanying the vascular pedicle. Disruption of these pathways may contribute to venous congestion, which remains a recognized cause of partial flap necrosis. Consequently, meticulous handling of the pedicle, preservation of venous structures where possible, and avoidance of compression or kinking during flap transfer are important technical considerations aimed at optimizing venous outflow and flap viability [6,12,15]. Studies have further demonstrated the intimate relationship between the PIA, posterior interosseous nerve, and surrounding vascular plexuses within the intermuscular septum between the extensor carpi ulnaris and extensor digiti minimi muscles [9,12,13]. Preservation of these structures during dissection is therefore essential not only to maintain adequate flap perfusion, but also to minimize the risk of neuropraxia and postoperative complications [9].

Anatomical variability in the number, calibre, and distribution of septo-cutaneous perforators has also been extensively described in the literature [4,6]. Most anatomical studies report between four and eight perforators arising along the course of the PIA, predominantly concentrated within the middle third of the forearm [6]. However, variations including hypoplastic vessels, absent distal communicating branches, and even congenital absence of the PIA have been reported and may significantly compromise reverse-flow flap perfusion [12,14]. These findings reinforce the importance of meticulous preoperative vascular assessment, particularly in patients with previous trauma, scarring, infection, or prior surgery [7,14]. Furthermore, evolving perforator-based concepts and modifications of the PIA flap continue to expand its reconstructive applications, highlighting the ongoing importance of detailed vascular anatomical knowledge in optimizing flap survival and functional outcomes [12,13,15].

Surgical technique 

The PIA flap is most commonly performed as a distally based, reverse-flow fasciocutaneous flap for coverage of dorsal hand and wrist defects. Preoperative planning includes careful assessment of the defect following adequate debridement and skeletal stabilization, with flap dimensions tailored to the size and location of the defect [15,16]. The central axis of the flap is typically marked along a line joining the lateral epicondyle of the humerus to the distal radioulnar joint, corresponding to the course of the PIA [15,17]. Cutaneous perforators are identified preoperatively using handheld Doppler, most commonly located at the junction of the proximal and middle thirds of the forearm, and the pivot point is usually set approximately 2-3 cm proximal to the distal radioulnar joint to preserve the distal anastomosis with the anterior interosseous artery [15,18].

Flap elevation is performed under tourniquet control, often without exsanguination to facilitate visualization of perforators, with dissection proceeding from distal to proximal along the intermuscular septum between the extensor carpi ulnaris and extensor digiti minimi [15,17]. The vascular pedicle is carefully preserved, including one or more septocutaneous perforators, and the flap may be raised as a fasciocutaneous, adipofascial, or osteocutaneous variant depending on reconstructive requirements [18,19]. Once elevated, the flap is transposed to the defect either through a subcutaneous tunnel or via an open pathway, ensuring that the pedicle is not kinked or compressed, as this may predispose to venous congestion [18,20]. The donor site is typically managed with split-thickness skin grafting, and technical modifications such as inclusion of additional perforators, alteration of skin markings, or incorporation of the recurrent branch of the PIA have been described to improve distal reach and reduce complications [15,17,19].

Indications 

The PIA flap is primarily indicated for soft tissue defects of the distal forearm, wrist, and dorsum of the hand, particularly when there is exposure of tendons, bone, or neurovascular structures requiring durable coverage [16,21]. It is especially useful for reconstruction of the dorsal hand, first web space, and thumb, where thin, pliable tissue is needed to preserve function and facilitate tendon gliding [19,21,22]. Common indications include trauma, infection, tumor excision, and post-burn contracture release, particularly of the first web space [21].

The PIA flap is advantageous when preservation of the radial and ulnar arteries is required, making it a suitable regional alternative to free flaps for moderate-sized defects with reduced operative time and morbidity [23]. However, its use is limited in very large defects or in cases with compromised posterior interosseous circulation, where free tissue transfer may be more appropriate [23,24].

The PIA flap is particularly valuable in post-traumatic hand and wrist defects, where exposed tendons, bones, joints, or neurovascular structures require vascularized coverage and local tissue options may be limited [25,26]. Its indications extend beyond trauma to include defects following tumor or sarcoma resection, where single-stage soft tissue coverage may support limb preservation and timely postoperative adjuvant therapy [27,28]. The flap is also useful for functional reconstruction of the thumb, first web space, and distal hand, including post-burn contracture release and selected thumb tip or fingertip defects requiring thin, pliable tissue [29,30]. In selected cases, the antegrade PIA flap may be considered for posterior elbow defects, particularly when robust but mobile coverage is required and other regional options carry greater donor-site morbidity [31].

Outcomes and complications 

The PIA flap has consistently demonstrated high reliability, with reported flap survival rates ranging from approximately 81% to 100% across clinical series [16,32,33]. Most studies report favorable functional and aesthetic outcomes, with stable soft tissue coverage, satisfactory tendon gliding, and good color and texture match to the dorsal hand [16,33]. Functional outcomes, including return to daily activities and acceptable disability scores, are generally favorable, particularly when early mobilization is achieved [16]. In addition, the flap offers low donor-site morbidity, with most patients reporting acceptable cosmetic outcomes and high satisfaction, despite the frequent need for skin grafting at the donor site [33,34].

Despite its reliability, the PIA flap is associated with a distinct complication profile, the most common being venous congestion, reported in up to 16-24% of cases [33,35]. This may lead to partial distal flap necrosis, which is typically managed conservatively or with secondary procedures such as debridement and skin grafting [16,35]. Total flap loss is uncommon but has been reported in a small proportion of cases, generally below 5% [34,35]. Additional complications include donor-site morbidity such as delayed wound healing, sensory disturbances, and scarring, as well as occasional neuropraxia of the posterior interosseous nerve [34,36]. Technical modifications, including careful pedicle handling, avoidance of tunnelling, and flap delay procedures, have been shown to reduce complication rates and improve overall outcomes [33,35].

Beyond flap survival, successful reconstruction using the PIA flap is closely related to restoration of hand function, preservation of tendon gliding, and early rehabilitation. Because the flap provides thin and pliable fascio-cutaneous tissue, it is particularly well suited for dorsal hand and wrist defects where excessive bulk may impair extensor tendon excursion and digital mobility [2,16,19]. Several studies have demonstrated satisfactory functional outcomes with preservation of hand motion, acceptable disability scores, and return to daily activities following reconstruction [16,21,33]. Early physiotherapy and splint-assisted rehabilitation are considered essential components of postoperative management, particularly in patients with associated tendon, joint, or skeletal injuries, as prolonged immobilization may contribute to stiffness and delayed functional recovery [2,20].

Venous congestion remains the most frequently encountered complication of the PIA flap and represents the principal cause of partial distal flap necrosis [6,19,35]. Multiple contributing factors have been described, including pedicle kinking, excessive tunnelling, tension during inset, compression from postoperative oedema, and injury to the subcutaneous venous plexus during flap elevation [6,15,19]. Anatomical variability of the distal communicating branch between the posterior and anterior interosseous arteries may further compromise retrograde perfusion and increase the risk of vascular insufficiency [13,14]. Several technical modifications have therefore been proposed to improve flap reliability, including preservation of a broad fascial pedicle, avoidance of tight subcutaneous tunnels, inclusion of additional perforators, flap delay procedures, and careful handling of the venae comitantes during dissection [15,17,35].

Donor-site morbidity following PIA flap harvest is generally considered acceptable, although the need for split-thickness skin grafting remains a recognized limitation when larger flaps are harvested [19,34]. Reported donor-site complications include delayed wound healing, hypertrophic scarring, sensory disturbances, cold intolerance, and occasional weakness related to extensive dissection near the posterior interosseous nerve [34-36]. Nevertheless, compared with radial forearm flap reconstruction, preservation of the radial and ulnar arteries remains a major advantage of the PIA flap and may reduce the risk of vascular compromise to the hand [1,21,37]. In addition, avoidance of microsurgical anastomosis shortens operative time and may make the flap particularly useful in resource-limited environments or in patients who are poor candidates for prolonged microsurgical procedures [2,23].

Despite the overall favorable outcomes reported in the literature, most available evidence regarding PIA flap reconstruction is derived from small retrospective case series with heterogeneous patient populations and variable outcome reporting [19,33,35]. Differences in flap design, perforator selection, postoperative rehabilitation protocols, and definitions of complications limit direct comparison between studies. Future prospective studies with standardized functional outcome measures and longer-term follow-up may help further define the optimal indications, complication profile, and comparative effectiveness of the PIA flap in modern upper limb reconstruction.

Comparison with other forearm flaps 

The PIA flap should be considered alongside other regional forearm-based options, particularly the radial forearm flap, ulnar artery-based flaps, radial artery perforator/adipofascial flaps, superficial palmar branch of radial artery flaps, and free perforator flaps from the ipsilateral forearm [21]. The radial forearm flap remains highly reliable and versatile for elbow, wrist, and hand reconstruction, with reported primary healing rates of approximately 95%; however, its main limitation is sacrifice of the radial artery and the potential for donor-site morbidity [34,37]. Reverse adipofascial radial forearm flaps may offer shorter operative time and fewer donor-site problems than PIA flaps in some dorsal hand defects, but they still require sacrifice of a major artery of the hand [38]. In contrast, the PIA flap preserves both radial and ulnar arteries, provides thin and pliable tissue, and remains useful for dorsal hand, wrist, thumb, and first web space reconstruction [21,33].

Other vessel-sparing alternatives include distal radial artery perforator flaps, distal ulnar artery perforator flaps, superficial branch of the radial artery (SUPBRA)-based flaps, and free posterior interosseous perforator flaps. These options can provide good like-for-like reconstruction and low donor-site morbidity, particularly for smaller or more distal digital defects [24,39]. Comparative forearm flap studies suggest that vessel-preserving reverse forearm flaps can achieve reliable coverage with acceptable functional outcomes and no major difference in Disability of the Arm, Shoulder, and Hand (DASH) or total active motion between flap types [40]. However, the PIA flap has the advantage of avoiding microsurgical anastomosis and remaining within the same operative field, while its main disadvantages are technical difficulty, anatomical variability, shorter distal reach, and venous congestion risk [35,41]. Therefore, flap choice should be individualized according to defect size, location, vascular status, need for glabrous or nonglabrous tissue, and available microsurgical expertise.

Recent comparative studies have further emphasized the role of the PIA flap as an effective regional option for medium-sized dorsal hand defects, particularly when thin and pliable tissue coverage with preservation of the major forearm vessels is desired [42,43]. Although free flaps such as the ALT flap provide greater tissue volume and versatility for extensive or composite defects, they are associated with longer operative times, increased technical demands, and potential flap bulkiness requiring secondary contouring procedure [44]. Alternative perforator-based options, including radial artery perforator, anterior interosseous artery perforator, and recurrent branch anterior interosseous artery propeller flaps, have also shown reliable outcomes in selected cases, particularly when the distal interosseous arterial communication is absent or compromised [45,46]. Furthermore, muscle-based flaps such as the anconeus flap remain valuable for contaminated wounds, osteomyelitis, tendon exposure, and first web-space reconstruction because of their robust vascularity and low donor-site morbidity, although modern reconstructive trends increasingly favor fascio-cutaneous and perforator-based flaps because of improved contour, tendon gliding, and aesthetic outcomes [47,48].

The evolution of perforator flap surgery has significantly influenced contemporary upper limb reconstruction by enabling thinner, more anatomically tailored reconstructions while minimizing donor-site morbidity and preserving major vascular axes [24,39,45]. In this context, the PIA flap remains relevant as a vessel-preserving regional option that bridges traditional axial-pattern flap concepts and modern perforator-based reconstructive principles [21,24]. Nevertheless, increasing availability of perforator and free-style flap techniques continues to expand the reconstructive armamentarium available for hand and wrist defects.

## Conclusions

The PIA flap remains a reliable and versatile option for reconstruction of soft tissue defects of the dorsal hand, wrist, and distal forearm, offering the key advantage of preserving the major vascular axes while providing thin, pliable, and well-vascularized coverage with satisfactory functional and aesthetic outcomes. Although associated with challenges such as venous congestion and anatomical variability, these limitations can be mitigated through careful surgical technique and appropriate patient selection. Positioned between local pedicled and free flap options, the PIA flap serves as an effective vessel-preserving solution for moderate-sized defects, maintaining an important role in contemporary upper limb reconstruction.
